# Prevalence of intellectual disability in New South Wales, Australia: a multi-year cross-sectional dataset by Local Government Area (LGA)

**DOI:** 10.1016/j.dib.2020.105673

**Published:** 2020-05-12

**Authors:** Phillippa Carnemolla, Preeyaporn Srasuebkul, Hamish Robertson, Julian Trollor, Nick Nicholas

**Affiliations:** a University of Technology Sydney; b Department of Developmental Disability Neuropsychiatry, School of Psychiatry, UNSW Sydney, Sydney, Australia

**Keywords:** Disability, Intellectual disability, Local government, Planning, Prevalence, Geography, Mapping

## Abstract

The presented dataset relates to a research project titled “My Home My Community” undertaken at University of Technology Sydney (UTS) which has been funded by the National Disability Insurance Agency (NDIA) Australia. The dataset reports estimated prevalence rates of Intellectual Disability in NSW by local government area (LGA) from 2010 – 2015. The dataset is a re-examination of a cohort of 92, 542 people with intellectual disability from a larger linked research dataset built by *the Department of Developmental Disability Neuropsychiatry, School of Psychiatry, UNSW*. The dataset in this paper is presented in a multi-year cross-sectional format.

The cohort of people with Intellectual Disability was analysed to estimate, quantify and visualise where people with intellectual disability live in New South Wales (NSW). The cohort analysed in this dataset had been generated in an earlier project undertaken by the UNSW-based authors. This dataset was generated to share with local governments in Australia and has the potential to be more widely used in a range of health policy and planning research, and city and regional planning research environments. It represents one of the only datasets currently available in Australia on Intellectual Disability describing prevalence rates at a local government area level. This dataset allows for population comparisons in other Australian states and internationally and can be examined in combination with other social and economic datasets to continue to build evidence about disability, planning and geography.

Specifications table

**Table:** 

Subject	Geography, Planning and Development
Specific subject area	Maps the prevalence rates of People with Intellectual Disability across Local Government Areas in NSW, Australia.
Type of data	Aggregate data of LGA population and estimated prevalence of people with intellectual disability. Format: Table (Microsoft Excel) Map (heat maps included in manuscript) Google Map data: (https://www.google.com/maps/d/u/0/viewer?mid=1jfh885FmRAgup-kDxu4qb2dpHvTPDyJt&ll=-32.21186728760618%2C146.7204319780418&z=7
How data were acquired	The data presented in this paper was acquired by extracting and aggregating data from a dataset of People with Intellectual Disability (ID) examining access to government health services in NSW Australia. The original cohort was generated by linking a collection of larger population datasets from the following government sectors: disability, health, corrective services and targeted specialist support services in public schools, Public Guardian and Ombudsman services to determine prevalence of People with ID in the population of NSW, Australia. The preceding project was conceived and developed by the the Department of Developmental Disability Neuropsychiatry, School of Psychiatry, UNSW with the cohort profile described in two research papers [1,2].
Data format	Aggregated Raw Data
Parameters for data collection	Data describing the cohort of People with Intellectual Disability had already been collected as part of larger dataset. The original data was re-analysed to screen by local government area as well as Intellectual disability status. It was then aggregated (under strict ethical requirements individual level data cannot be shared) to population at an LGA level. Ethical approval was obtained from the NSW Population and Health Services Research Ethics Committee (AU RED Study Reference Number: HREC/13/CIPHS/7; CINSW Reference Number: 2013/02/446). All data is presented as aggregated in line with requirements stated in the CINSW HREC Approval Number 2013/02/446,(2018) Protocol *Improving inclusion for people with intellectual and developmental disability in their community: Improving mainstream service delivery by local governments.*
Description of data collection	The data was collected by extracting data on population numbers and local government area of home address from a larger dataset of a cohort of People with Intellectual Disability which is stewarded by the Department of Developmental Disability Neuropsychiatry, School of Psychiatry, UNSW. Extracted data was aggregated to reveal prevalence rates of People with ID across all Local Government areas in NSW.
Data source location	1. University of Technology Sydney Faculty of Design Architecture and Building School of Built Environment Sydney, NSW Australia 2. University of New South Wales (UNSW) Department of Developmental Disability Neuropsychiatry, School of Psychiatry, Sydney, NSW, Australia
Data accessibility	Hosted with the article

## Value of the data

• One of the difficulties of analysing prevalence rates of Intellectual Disability in Australia and nationally has been a lack of consensus across jurisdictions on how Intellectual Disability is defined and captured in larger datasets. This dataset is drawn from a population cohort that can be considered more accurate than other Australian datasets for a number of reasons; Firstly, it was generated by linking a number of large databases; Secondly, it captures secondary diagnoses of Intellectual Disability and thirdly, it is able to isolate intellectual disability status from more broad disability or mental health status.
• Data on where People with Intellectual Disability (ID) live has not previously been able to be presented or compared at a Local Government Area (LGA) level in Australia. The distribution of people with ID has been limited to a single prevalence rate across larger National or State levels. This data represents a state-wide dataset for NSW Australia that details service access at the local government level.
• Researchers and policy-makers working in the fields of Disability, Local Government, Housing, Planning, Geography, Health and Inclusion. Local Governments across NSW Australia (128) can access it to understand the data in terms of their own LGA – to plan for services and preparing Disability Inclusion Action Plans (DIAPS). More broadly, local governments across Australia and internationally can interpret aspects of the data in their own local government context.
• Prevalence rates of People with Intellectual Disability can be very difficult to determine for a number of reasons. Firstly, because of the wide variance in level of intellectual disability (mild or severe). Secondly, data may not isolate Intellectual disability from disability in general. Thirdly, data may cluster intellectual disability with mental health diagnoses.
• data can be linked with other available datasets that sort by Local Government Area, to provide insight into what factors influence where people with intellectual disability live and why. This dataset represents aggregated data from one of the largest available cohorts of people with intellectual disability internationally.

## 1 Data Description

The dataset is a re-examination of a cohort of people with intellectual disability. The cohort links several administrative datasets from health, disability, justice and education service providers to identify people in NSW with neuropsychiatric disorders. The original data linkage precedes this published dataset and was undertaken by the Department of Developmental Disability Neuropsychiatry, School of Psychiatry, UNSW with the cohort profile described in two research papers [1,2]. Linkage of the data sets was performed by the NSW Centre for Health Record Linkage (CHeReL). The CHeReL links health-related data in NSW in accordance with State and Commonwealth ethical, legal, privacy and confidentiality requirements.

## 2 About the cohort

**How Intellectual Disability (ID) was identified in the databases:** All people identified as having ID either had a classification code for ID based on the Diagnostic and Statistical Manual of Mental Disorders (DSM) IV or had a diagnosis of intellectual disability by International Statistical Classification of Diseases and Related Health Problems (ICD-10) in their health record.

**Geographic location**: New South Wales Australia

Table One (supplied separately) shows the number of people with Intellectual disability living in each local government area in New South Wales, Australia, across a range of years 2010- 2015. Please note that a blank cell indicates that 0-5 people with intellectual disability were reported in the corresponding Local Government Area. Ethics requirements restrict reporting exact population numbers where cell values are less than five.

## 3 Google Maps link to geographic data

The most recent dataset for years 2014/5 has been visualised in the following Google Maps link: https://www.google.com/maps/d/u/0/viewer?mid=1jfh885FmRAgup-kDxu4qb2dpHvTPDyJt&ll=-32.21186728760618%2C146.7204319780418&z=7

Tables 1 and 2 below are supplementary tables that show the age distribution and percentage male/female for the 2010-2015 cohort, and the age distribution for the 2014/5 year data.

**Table 1:** Age Distribution and Percentage Male and Female across entire 2010-2015 population cohort of People with ID.

Age group	Male	%Male	Female	%Female	Total	%Total
0-4	253085	6.8	239543	6.3	492628	6.6
05-09	244956	6.6	231606	6.1	476562	6.3
10-14	229477	6.2	216664	5.7	446141	5.9
15-19	237909	6.4	224854	5.9	462763	6.2
20-24	264497	7.1	254076	6.7	518573	6.9
25-29	273426	7.3	273617	7.2	547043	7.3
30-34	270173	7.3	272223	7.2	542396	7.2
35-39	247442	6.6	250185	6.6	497627	6.6
40-44	259187	7.0	267191	7.0	526378	7.0
45-49	237000	6.4	245570	6.5	482570	6.4
50-54	247862	6.7	254503	6.7	502365	6.7
55-59	225766	6.1	233599	6.2	459365	6.1
60-64	201207	5.4	207778	5.5	408985	5.4
65+	533990	14.3	618677	16.3	1152667	15.3
**Grand Total**	**3725977**		**3790086**		**7516063**	

Note: %Male, %Female and %Total are percentages of each age group over Grand Total.

**Table 2:** Age Distribution 2014/5.

Age group	ID Male	%ID Male	ID Female	%ID Female	Total	% ID Total
0-4	1629	3.3	1097	3.5	2727	3.4
05-09	6623	13.5	3251	10.2	9879	12.2
10-14	8832	18.0	4249	13.4	13084	16.2
15-19	6582	13.4	3543	11.2	10132	12.5
20-24	5007	10.2	3266	10.3	8279	10.2
25-29	3878	7.9	2688	8.5	6573	8.1
30-34	2646	5.4	2087	6.6	4740	5.9
35-39	2311	4.7	1794	5.7	4110	5.1
40-44	2289	4.7	1869	5.9	4165	5.1
45-49	2110	4.3	1628	5.1	3743	4.6
50-54	2051	4.2	1599	5.0	3656	4.5
55-59	1572	3.2	1456	4.6	3033	3.7
60-64	1462	3.0	1223	3.9	2686	3.3
65+	2170	4.4	1974	6.2	4150	5.1
**Grand Total**	**49163**		**31725**		**80960**	

Note 1: %Male, %Female and %Total are percentages of each age group over Grand Total

Note 2: Grand total of total includes people with invalid age group and sex

**Fig. 1:**
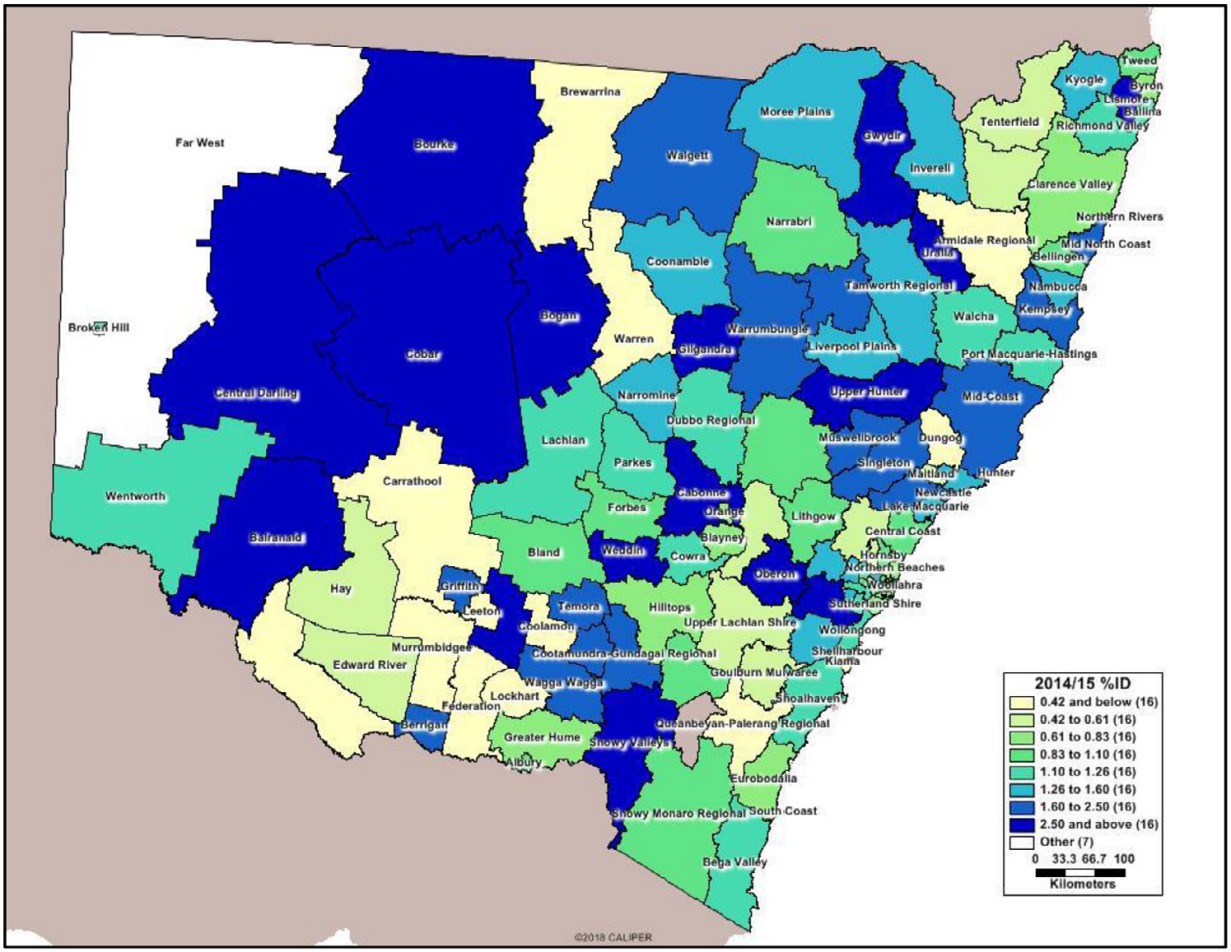
2014/15 prevalence rates of Intellectual disability by LGA. NSW, Australia.

**Fig. 2:**
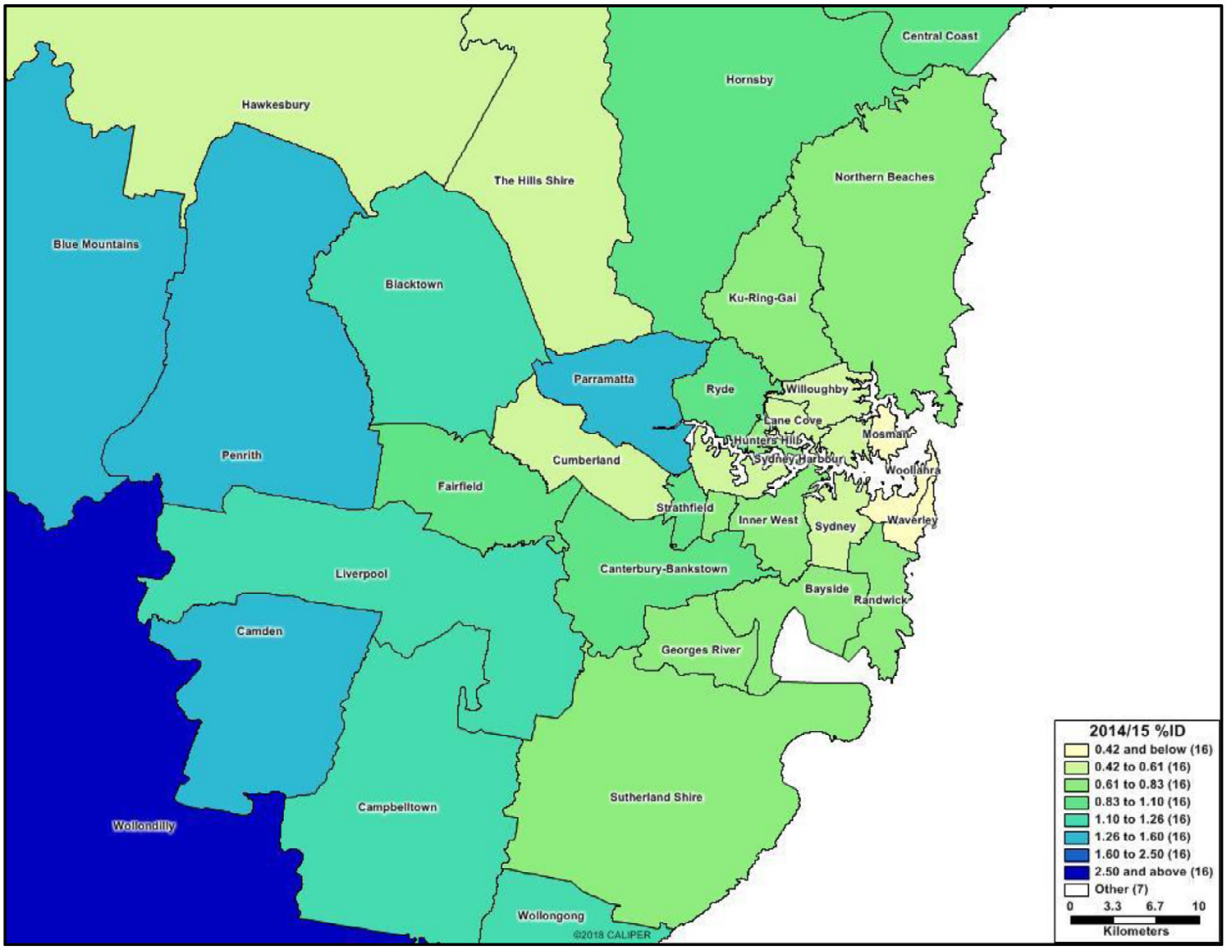
Metropolitan Sydney Area Detail of 2014/15 prevalence rates of Intellectual disability by LGA. NSW, Australia.

Fig. 1 below is a map showing prevalence rates of Intellectual disability in each Local Government Area. Fig. 2 is a map showing the detail view of the Sydney Metropolitan Area – the most densely populated area of NSW.

## 4 Experimental Design, Materials, and Methods

The cohort of people with Intellectual Disability living in NSW was derived from a larger funded project [1] led by 3DN UNSW. The dataset links a suite of smaller administrative datasets across government and other registries, identifying people with neuropsychiatric disorders in NSW, including those with Intellectual Disability. Person level data was obtained from:

• NSW Admitted Patient Data Collection (APDC)
• NSW Emergency Department Data Collection (EDDC)
• NSW Mental Health Ambulatory (MH-AMB) Data Collection
• NSW Registry of Births Deaths & Marriages
• NSW Ombudsman
• NSW Public Guardian data set
• Statewide Disability Services (SDS) from NSW Corrective Services
• Targeted Specialist Support Services from NSW Department of Education.

The raw linked database contained a large number of individuals in NSW with neuropsychiatric disorders (n: 2,097,017) from which a sub-cohort of people with Intellectual Disability were identifiable according to diagnostic codes. The data is bound by the CINSW HREC Approval Number 2013/02/446 Protocol *Improving inclusion for people with intellectual and developmental disability in their community: Improving mainstream service delivery by local governments.*

Aggregated raw data related to people with intellectual disability can be shared publicly and tells a story about how many people live with Intellectual Disability in NSW and the Local Government Areas where they live. Using SPSS software, this cohort data was analysed by the Local Government Area according to an individual's registered home address. These fields of data were available for 5 years from 2010-2015. Geographic representations of prevalence in each LGA in NSW were visualised using Power BI software.

## Appendix Supplementary materials

Image, application 1

Image, application 2
